# Effects of Salinity on Assembly Characteristics and Function of Microbial Communities in the Phyllosphere and Rhizosphere of Salt-Tolerant *Avicennia marina* Mangrove Species

**DOI:** 10.1128/spectrum.03000-22

**Published:** 2023-02-06

**Authors:** Xiangxia Yang, Zhian Dai, Rongwei Yuan, Zhenhua Guo, Hanxiao Xi, Zhili He, Mi Wei

**Affiliations:** a School of Agriculture, Shenzhen Campus of Sun Yat-sen University, Shenzhen, Guangdong, China; b Environmental Microbiomics Research Center, School of Environmental Science and Engineering, Southern Marine Science and Engineering Guangdong Laboratory (Zhuhai), State Key Laboratory of Biocontrol, Sun Yat-sen University, Guangzhou, China; University of Massachusetts Amherst

**Keywords:** *Avicennia marina*, phyllosphere, rhizosphere, salinity, microbial communities

## Abstract

It is of great significance to explore the structure and salinity response of microbial communities in salt-tolerant plants to understand the mechanisms of plant-microbe interactions. Herein, we investigated the phyllosphere and rhizosphere microbial communities of Avicennia marina, a pioneer salt-tolerant plant, at three sites with different salinities in the coastal intertidal zone. The results showed that salinity had different effects on phyllosphere and rhizosphere microbial communities and had a greater impact on bacterial communities and bacterial network interactions. The rhizosphere bacterial community alpha diversity significantly increased with increasing salinity. Moreover, the relative abundance of *Proteobacteria* decreased significantly, while that of *Bacteroidota* and *Actinobacteriota*, with stronger salt tolerance and nutrient utilization capacity, increased significantly. Functional prediction indicated that the microbial communities could produce catalase, peroxidase, 3-phytase, and tryptophan synthase, which may exert potential antistress and growth-promoting functions. Among them, catalase, 3-phytase, alkaline phosphatase, and acid phosphatase increased significantly in the phyllosphere and rhizosphere bacterial communities and the phyllosphere fungal community with increasing salinity. Importantly, the dominant taxa *Kushneria* and *Bacillus*, which are salt tolerant and growth promoting, were isolated from the phyllosphere and rhizosphere, respectively, and verified to have the ability to alleviate salt stress and promote the growth of rice.

**IMPORTANCE** *Avicennia marina* is a pioneer salt-tolerant plant in coastal intertidal mangroves, an efficient blue carbon ecosystem. It is of great importance to explore how salinity affects the phyllosphere and rhizosphere microbial communities of *A. marina.* This study showed that the microbial communities in the phyllosphere and rhizosphere of *A. marina* had different constitutive properties, adaptive network interactions, and potential stress-promoting functions. Furthermore, the dominant bacteria *Kushneria* and *Bacillus* were obtained from the phyllosphere and rhizosphere, respectively, and their coculture with rice could effectively alleviate salt stress and promote rice growth. Additionally, the effects of salinity changes on microbial community structure, associations, and functional potential in the phyllosphere and rhizosphere of *A. marina* were observed. This study has enriched our understanding of the microbial community structure, function, and ecological stability of mangrove species in coastal intertidal zones and has practical significance for improving crop yield by using salt-tolerant plant microbiomes.

## INTRODUCTION

Mangrove forests growing in the intertidal zone of tropical and subtropical coasts are wetland woody plant communities, which are a special ecosystem distributed in the land-sea interface (1). Mangrove ecosystems play an important role in biodiversity maintenance, wind protection, dike fixation, carbon storage, and seawater purification (2). Mangrove soil has a high salinity due to periodic tidal seawater immersion and intense biological salinization of mangroves (3). Mangrove plants have grown in this habitat for a long time and have specialized a set of adaptive mechanisms different from those of terrestrial or freshwater plants (4). Avicennia marina belongs to *Avicennia* of the Verbenaceae, which has developed finger-like respiratory roots and leaves with salt glands for salt secretion functions, and it is known as the pioneer of mangroves due to its strong salt tolerance (5).

Plants are colonized by a large number of microbial species, including bacteria, fungi, archaea, and many protists, which play important roles throughout the life cycle of plants and have a positive impact on host health, growth, and productivity (6). A previous study indicated that complex and dynamic interactions have been established between salt-tolerant plants and associated microbiota during long-term coevolution (7). The microbiota inhabiting the different spatial niches of these plants have important roles in enhancing host resistance to salt stress and promoting productivity (8, 9). It is necessary and meaningful to study the formation, structure, and function of microbial communities in the phyllosphere and rhizosphere of *A. marina* with salt gland structure and special respiratory roots.

Previous studies have shown that the rhizosphere of salt-tolerant plants is enriched with a large number of microorganisms related to salt stress adaptation (10, 11). The microbiota in rhizosphere soils of salt-tolerant plants have a greater abundance of genes that promote plant growth and alleviate salt stress than the microbiota in bulk soils (7). In addition, it has been suggested that salinity has negative effects on the soil and root microbiome of spinach, resulting in taxonomic and functional differences between them, but has no effect on the phyllosphere microbiome (12). However, it is not clear how salinity affects the rhizosphere microbial community of *A. marina*, which grows in coastal mangrove ecosystems with different salinities. Some studies have shown that with the increase in rhizosphere salinity, the ability of leaves to secrete salt is enhanced and the phyllosphere salinity is increased simultaneously (13). We hypothesized that changes in rhizosphere salinity might also affect the composition and function of phyllosphere microbial communities in *A. marina.* It will be interesting to explore the differences in the effects of salinity on microbial communities in the phyllosphere and rhizosphere.

It is also valuable to identify the important microbial resources that play an important role in salt-tolerant growth promotion of *A. marina*. Strikingly, the potential utilization of these microbial resources in salt-tolerant growth promotion of important crops such as rice is promising. Pseudomonas maricaloris, isolated from the rhizosphere of *A. marina*, showed high ACC (1-aminocyclopropane-1-carboxylate) deaminase activity. It could reduce the endogenous level of ACC in mangrove seedlings under salt stress and improve plant performance (14). However, among the culturable microorganisms in the phyllosphere and rhizosphere, what other microbial groups can assist the host plants in salt tolerance and growth promotion? Is this feature universal?

In this work, we sought to explore the phyllosphere and rhizosphere microbial communities and important culturable species of *A. marina* in three different soil salinities, focusing on the following objectives: (i) identify how salinity affects microbial community structure in the phyllosphere and rhizosphere of *A. marina*, (ii) determine the adaptive adjustment of phyllosphere and rhizosphere microbial functions to changes in salinity, and (iii) identify the important microbial resources that may play an important role in salt-tolerant growth promotion of *A. marina* or other crops and to verify their functions.

## RESULTS

### Influences of environmental factors on the rhizosphere microbial communities.

After quality filtering, a total of 1,066,173 and 1,172,826 bacterial and fungal sequences, respectively, were obtained and then clustered into operational taxonomic units (OTUs) at a 97% sequence identity level. The sequence numbers of bacteria and fungi in each sample were normalized to 26,159 and 16,801, respectively. Basic information on the sequencing results, which could be used for subsequent analysis, is shown in Table S1 and Fig. S1 in the supplemental material.

**FIG 1:**
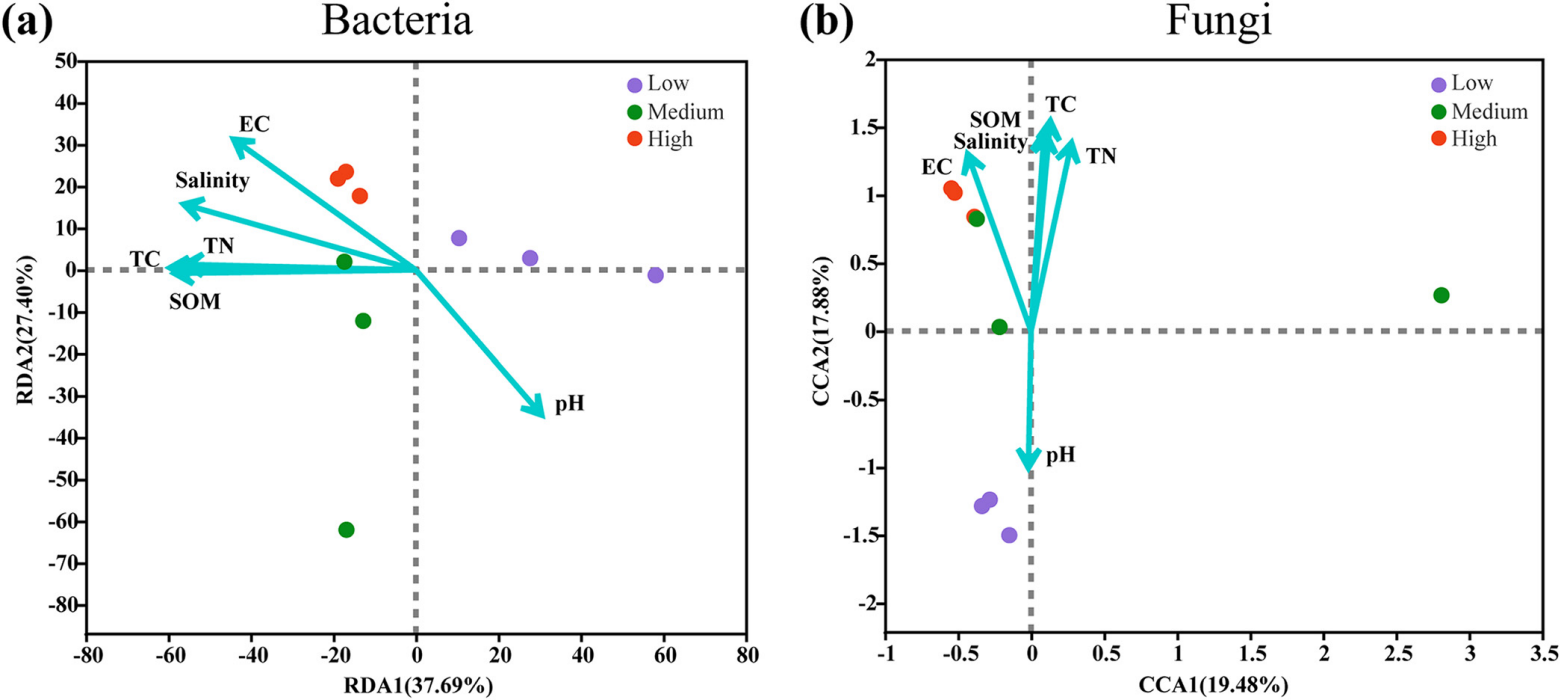
RDA showing the linkage between soil chemical properties in the rhizosphere bacterial community (a) and CCA showing the linkage between soil chemical properties in the rhizosphere fungal community (b) at the OTU level.

The physicochemical parameters of rhizosphere soil samples from three sampling sites were analyzed to assess the environmental conditions of rhizosphere microbial communities (Table S2). In this study, soil salinity was verified by testing both soil electrical conductivity (EC) and *in situ* salinity. There was a great difference in EC values and *in situ* salinities among the three sampling areas (Table S2). The EC value was the highest in Taiping, in the middle in Leizhou, and the lowest in Shenzhen. According to the different EC levels, the three sampling sites were defined as high-, medium-, and low-salinity groups. We conducted redundancy analysis (RDA) and canonical-correlation analysis (CCA) to understand the relationship between rhizosphere microbial communities and environmental factors (Fig. 1). The RDA and CCA results explained 65.09% and 37.36% of the variation in the rhizosphere bacterial and fungal communities, respectively (Fig. 1a and b). The results showed that EC, total nitrogen (TN), soil organic matter (SOM), salinity, and total carbon (TC) are important factors that influence bacterial and fungal communities, except for pH (Fig. 1). The results generally agreed with the Mantel test results, indicating that except for the pH value, the soil physicochemical factors were significantly correlated with the rhizosphere bacterial and fungal community structures (*P < *0.05) (Table S3). Among them, soil EC had a high correlation coefficient, with that of the bacterial community the greatest (*r* = 0.6105) and that of the fungal community second only to TN (*r* = 0.5284). Therefore, we then focused on the effects of three different salinities on the microbial communities and functions of the phyllosphere and rhizosphere.

### Effects of salinity on microbial diversity in the phyllosphere and rhizosphere.

**FIG 2:**
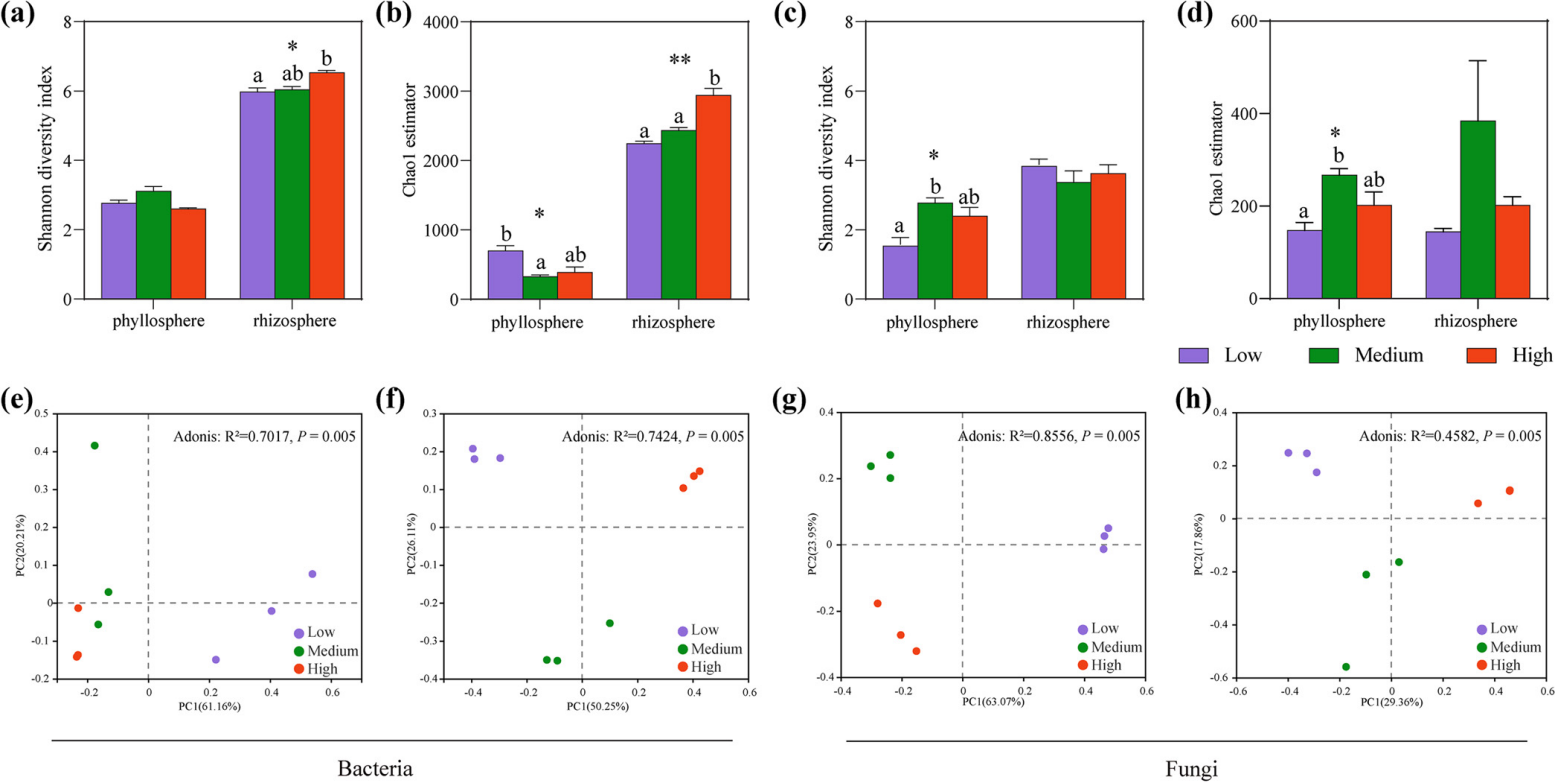
Diversity of microbial communities in the phyllosphere and rhizosphere with low, medium, and high salinity. (a to d) Alpha diversity of microbial communities of the phyllosphere and rhizosphere in *A. marina*: bacterial Shannon index (a), fungal Shannon index (b), bacterial Chao1 estimator (c), and fungal Chao1 estimator (d). Data are the mean ± standard error (*n* = 3). Different lowercase letters indicate significant differences (ANOVA, Tukey’s HSD test; *P < *0.05) among the three salinity gradients. *, *P < *0.05; **, *P* < 0.01; ***, *P* < 0.001. (e to h) PCoA of the phyllosphere and rhizosphere bacteria (e and f) and fungi (g and h) for three salinity gradients plotted based on the Bray-Curtis distance.

The rhizosphere bacterial community alpha diversity significantly increased with increasing salinity. It first increased and then decreased, which was not significantly different in the phyllosphere (Fig. 2a and b). Moreover, the richness of the bacterial community in the phyllosphere was the highest at low salinity (Fig. 2b). In contrast, the change in salinity had a significant effect on the phyllosphere fungal community, and the diversity and richness were the highest in the middle-salinity group. However, the change in salinity had no significant effect on the alpha diversity of the rhizosphere fungal community (Fig. 2c and d). This result suggests that only the alpha diversity of rhizosphere bacteria and phyllosphere fungi was significantly affected by salinity.

In addition, principal-coordinate analysis (PCoA) and Adonis based on Bray-Curtis distance were used to analyze the similarity and heterogeneity of bacterial and fungal communities under the salinity gradient (Fig. 2e to h). The results showed that the microbial communities in the phyllosphere and rhizosphere were obviously separate at different salinities, and they were significantly affected by salinity (*P = *0.005). Overall, salinity had different degrees of influence on microbial community diversity in the phyllosphere and rhizosphere, especially on the bacterial community.

### Effects of salinity on dominant taxa of microbial communities in the phyllosphere and rhizosphere.

**FIG 3:**
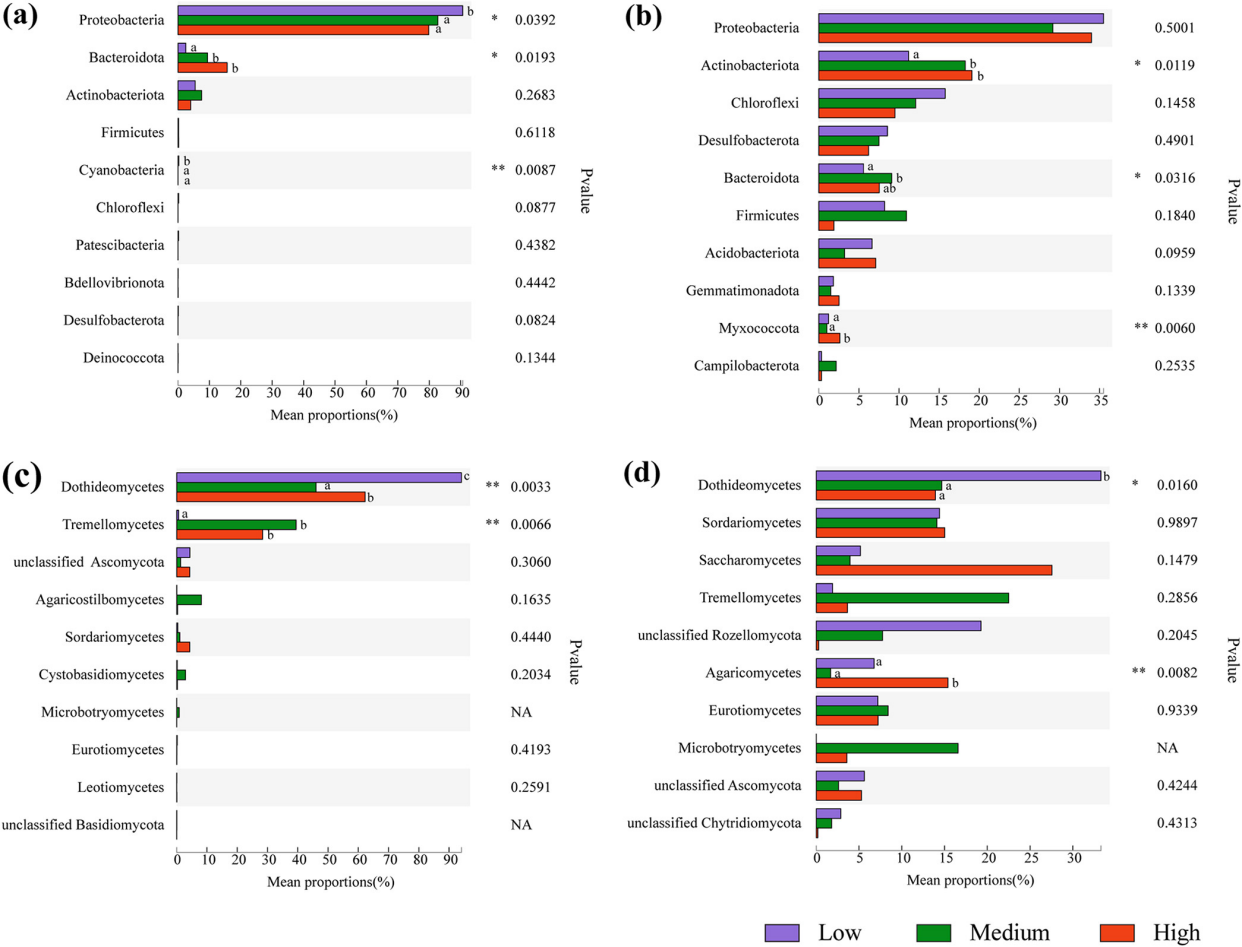
Top 10 relative abundances of bacterial taxa at the phylum level and of fungal taxa at the class level under three salinity gradients. (a) Phyllosphere bacteria; (b) rhizosphere bacteria; (c) phyllosphere fungi; (d) rhizosphere fungi. The *x* axis represents the average relative abundance (*n* = 3) in different groups of species, and the columns of different colors represent different groups. Different lowercase letters indicate significant differences (ANOVA, Tukey’s HSD test; *P < *0.05) among the three salinity gradients. On the far right is the *P* value: *, *P < *0.05; **, *P* < 0.01; ***, *P* < 0.001. NA, not available, indicates that the taxa cannot be detected in some groups.

The relative abundance changes of the dominant species (top 10) in the phyllosphere and rhizosphere microbial communities at the phylum (class) (Fig. 3) and genus levels (Fig. S2) were examined under different salinities. For the bacterial community, the relative abundance of *Proteobacteria* and *Cyanobacteria* decreased significantly with increasing salinity, while *Bacteroidota* increased significantly in the phyllosphere (Fig. 3a). The dominant phyla of rhizosphere bacteria and their trends were different; the relative abundance of *Actinobacteriota* and *Myxococcota* increased significantly with increasing salinity, while *Bacteroidota* reached the highest relative abundance at midsalinity (Fig. 3b). At the genus level, the relative abundance of *Maribius* decreased significantly with increasing salinity, while that of *Zunongwangia* increased significantly (Fig. S2a). Unlike with the phyllosphere, *Methyloceanibacter* reached the highest relative abundance at midsalinity in the rhizosphere (Fig. S2b). This result indicated that the effects of salinity on the dominant taxa of the bacterial community were significantly different between the phyllosphere and rhizosphere.

For the fungal community, two classes were significantly affected by salinity in the phyllosphere and rhizosphere (Fig. 3c and d). The relative abundance of Dothideomycetes was highest at low salinity and decreased with increasing salinity. In contrast, the relative abundance of Tremellomycetes increased significantly with increasing salinity in the phyllosphere, and Agaricomycetes reached the highest relative abundance at high salinity in the rhizosphere (Fig. 3d). At the genus level, the relative abundance of *Cladosporium* was highest at high salinity, *Neodevriesia* was highest at middle salinity, and *Hortaea* decreased significantly with increasing salinity in the phyllosphere (Fig. S2c). However, there was no significant change in the top 10 fungal taxa under the three salinity gradients in the rhizosphere. Overall, these results indicate that the salinity disturbance to the phyllosphere microbial communities was greater than that to the rhizosphere (Fig. S2d). The response of dominant groups to salinity is an adaptive strategy to maintain the stability of microbial communities in different ecological niches and may play a certain role in host salt resistance.

The leaf surface microstructure and colonization of microorganisms were observed by scanning electron microscopy (SEM). The gland structures on the leaves of *A. marina* can be seen in an SEM image (Fig. S3). Helical actinomycetes, spherical and rod-shaped bacteria, and fungi are distributed around the entrance of salt glands and on leaf surfaces (Fig. S3). It has also been observed that the hyphae of fungi and actinomycetes can penetrate salt glands to obtain nutrients and water from the host. Meanwhile, we found that the colonization of spheroidal and rod-shaped bacteria on the leaf surface of *A. marina* did not change significantly with increasing salinity, while the colonization of fungi around the salt glands was more frequent at medium and high salinity than at low salinity (Fig. S3), which was consistent with the results of the alpha diversity of the phyllosphere bacteria (Fig. 2a and b) and fungi (Fig. 2c and d) based on the sequencing results.

### Effects of salinity on microbial community network structure in the phyllosphere and rhizosphere.

**FIG 4:**
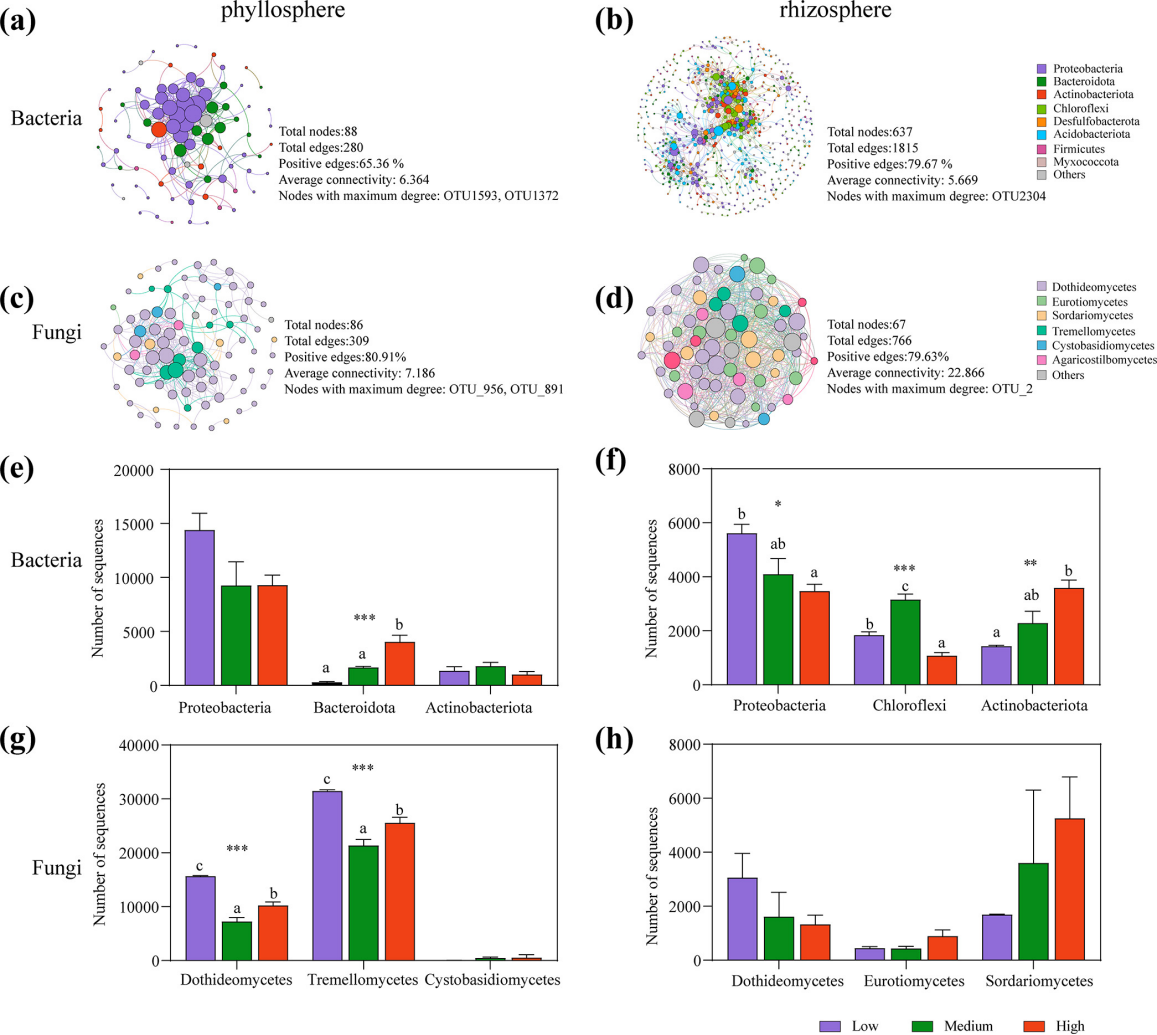
(a to d) Networks and topological properties of bacteria and fungi in the phyllosphere and rhizosphere of *A. marina*: phyllosphere bacteria (a), rhizosphere bacteria (b), phyllosphere fungi (c), and rhizosphere fungi (d). With the node size proportional to node connectivity, the node color represents various phyla or classes. (e to h) Changes in the absolute abundance of the dominant highly connected phylum or class in the network under three salinity gradients: phyllosphere bacteria (e), rhizosphere bacteria (f), phyllosphere fungi (g), and rhizosphere fungi (h). Data are the mean ± standard error (*n* = 3); different lowercase letters indicate significant differences (ANOVA, Tukey’s HSD test; *P < *0.05) among the three salinity gradients. *, *P < *0.05; **, *P* < 0.01; ***, *P* < 0.001.

Network analyses were performed based on bacterial and fungal OTUs to further explore potential microbial interactions and differences between the phyllosphere and rhizosphere under the three salinity gradients (Fig. 4). This result showed that more diverse groups participated in the interaction of the rhizosphere bacterial network than in the phyllosphere. *Proteobacteria*, *Bacteroidota*, and *Actinobacteriota* were mainly detected in the phyllosphere bacterial network (Fig. 4a). In addition to these three phyla, *Chloroflexi*, *Firmicutes*, *Acidobacteriota*, and *Myxococcota* were also detected in the rhizosphere bacterial network (Fig. 4b). Six classes were mainly detected in the fungal network, including Dothideomycetes, Tremellomycetes, Eurotiomycetes, Sordariomycetes, Cystobasidiomycetes, and Agaricostilbomycetes (Fig. 4c and d). Connectivity, also known as node degree, represents the strength of the connection between every two nodes. By studying the bacterial phyla and fungal classes with the highest connectivity, which are generally considered to be the keystone taxa in a network, we found that the abundance of phyla or classes occupying the keystone position in the network changes at different salinities. In the phyllosphere bacterial network, that most significantly affected by salinity was *Bacteroidota*, which increased with increasing salinity (Fig. 4e). *Proteobacteria*, *Chloroflexi*, and *Actinobacteriota* in the rhizosphere bacterial network were affected by salinity differently; the abundance of *Proteobacteria* decreased while *Actinobacteriota* increased with increasing salinity (Fig. 4f). In addition, Dothideomycetes and Tremellomycetes were the two classes that participated in the interaction network with the highest abundance at low salinity (Fig. 4g and h). For the rhizosphere fungal network, salinity changes had no significant effect on the three main groups involved in the interaction. Additionally, we explored the effects of different salinities on the highly interconnected hub microbial taxa known as “nodes of maximum degree” in the phyllosphere and rhizosphere communities. The abundance of nodes of maximum degree of OTU1593 (*Maribius*) of the phyllosphere bacterial network decreased with increasing salinity, whereas that of OTU1372 (*Micavibrionaceae*) increased. The nodes of maximum degree of OTU2304 (*Actibacter*) of the rhizosphere bacterial network decreased with increasing salinity, although there was no significant effect (Fig. S4). Salinity had no significant effect on the nodes of the fungal network, with maximum degrees of OTU_956 (Capnodiales), OTU_891 (Cryptococcus), and OTU_2 (*Rhodotorula sphaerocarpa*) (Fig. S4). In conclusion, both keystones and hubs are more susceptible to salinity in bacterial networks than in fungal networks.

### Effects of salinity on potentially important functions of the microbial communities in the phyllosphere and rhizosphere.

**FIG 5:**
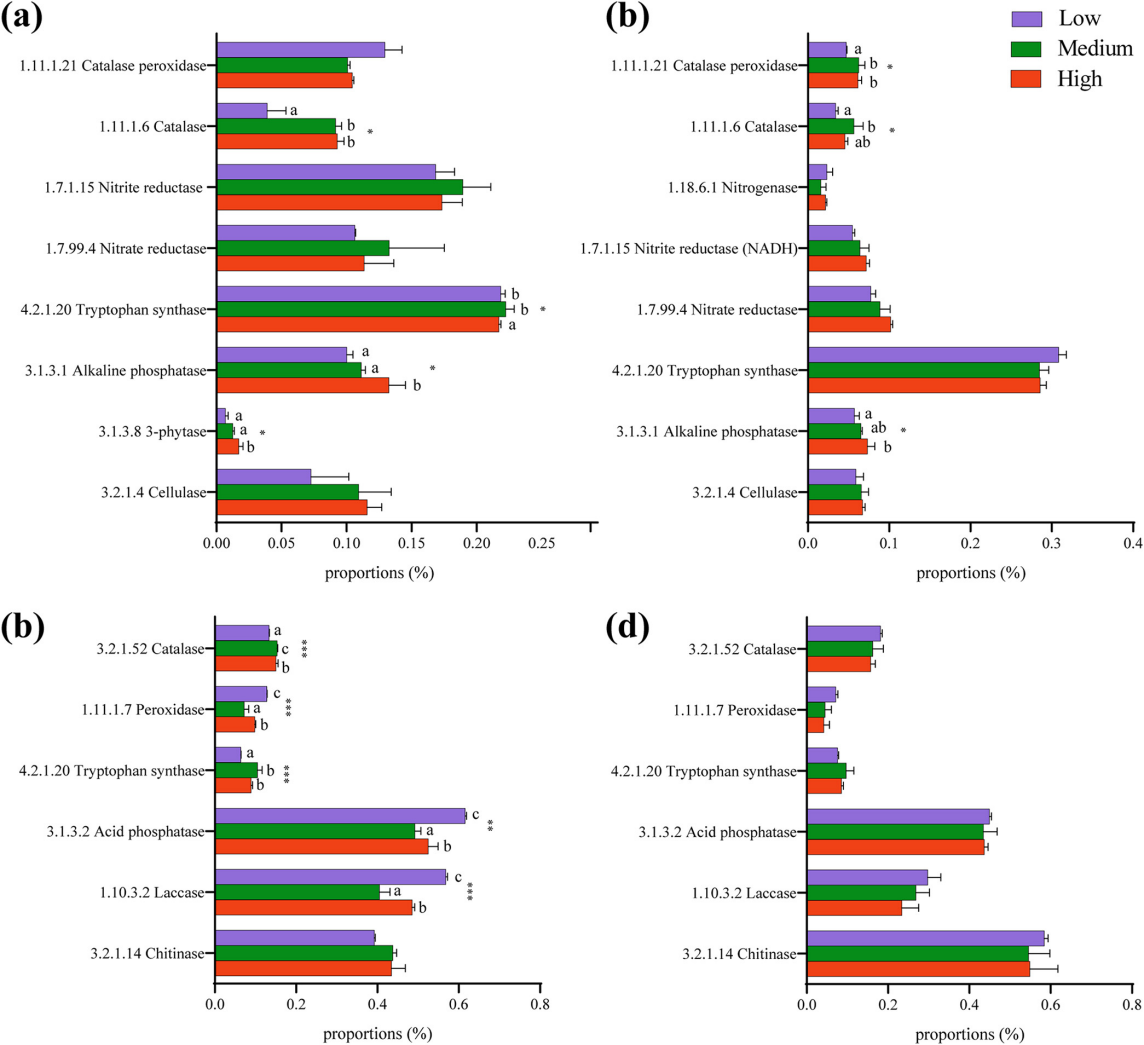
PICRUSt2 predicts the relative abundance of enzymes associated with promoting nutrient cycling and assisting plant stress resistance in microbial communities under three salinity gradients. (a) Phyllosphere bacteria; (b) rhizosphere bacteria; (c) phyllosphere fungi; (d) rhizosphere fungi. Data are the mean ± standard error (*n* = 3). Different lowercase letters indicate significant differences (ANOVA, Tukey’s HSD test; *P < *0.05) among the three salinity gradients. *, *P < *0.05; **, *P* < 0.01; ***, *P* < 0.001.

Functional prediction was used to detect changes in potential enzymes that promote nutrient cycling and assist plant stress resistance in the phyllosphere and rhizosphere microbial communities at different salinities (Fig. 5). With increasing salinity, the function of the microbial community in the phyllosphere and rhizosphere changed adaptively. The abundance of enzymes associated with phosphorus utilization (3-phytase and alkaline phosphatase) and enzymes assisting plant resistance to stress (catalase) increased in the phyllosphere and rhizosphere bacterial communities (Fig. 5a and b). In contrast to the bacterial community, the abundance of catalase and tryptophan synthase in the phyllosphere fungal community increased significantly with increasing salinity, but the abundance of these enzymes in the rhizosphere did not change significantly (Fig. 5c and d).

### Screening of functional microorganisms and analysis of their growth-promoting effects on rice.

**FIG 6:**
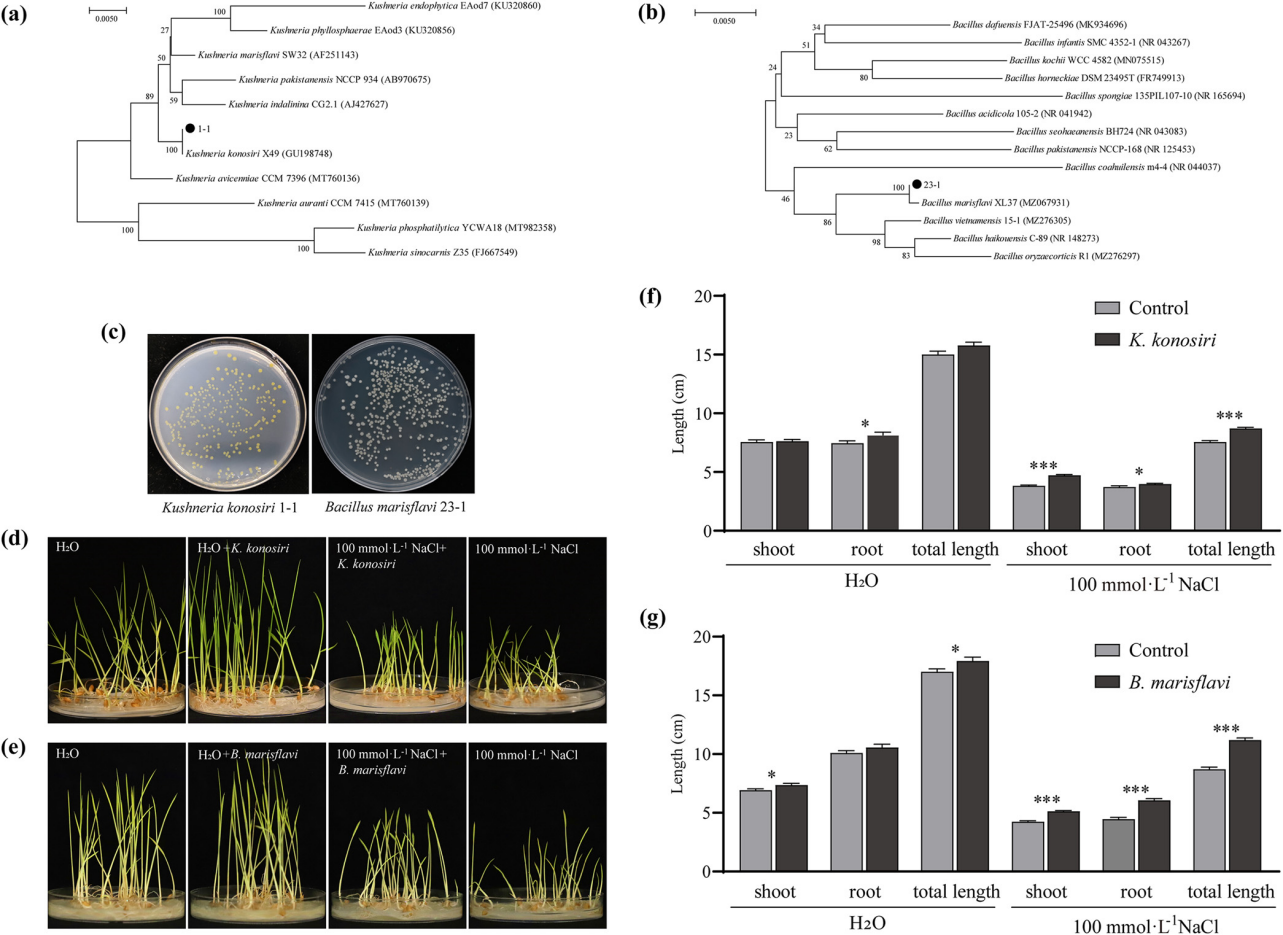
Effect of important microorganisms on the growth of rice seedlings under salt stress. (a to c) 16S rRNA phylogenetic tree of bacterial strain 1-1 (a) and strain 23-1 (b) and colony morphology of *K. konosiri* 1-1 and *B. marisflavi* 23-1 on R2A medium (c). (d) *K. konosiri* 1-1 growth-promoting phenotype; (e) *B. marisflavi* 23-1 growth-promoting phenotype; (f and g) growth-promoting effect of *K. konosir* 1-1 (f) and *B. marisflavi* 23-1 (g) on rice seedling treatments. Data are the mean ± standard error (*n* = 80). Different lowercase letters indicate significant differences (Student's *t* test, *P < *0.05). *, *P < *0.05; **, *P* < 0.01; ***, *P* < 0.001.

We obtained 56 strains of bacteria and 15 strains of fungi from the rhizosphere and 42 strains of bacteria and 10 strains of fungi from the phyllosphere. The results showed that more than 50% of the strains were more or less capable of dissolving phosphorus, fixing nitrogen, dissolving potassium, and tolerating and reducing salt. Among them, Kushneria konosiri 1-1 had a strong ability to dissolve organic phosphorus and fix nitrogen and Bacillus marisflavi 23-1 could dissolve both organic and inorganic phosphorus well. The results of high-throughput sequencing showed that the relative abundances of *Kushneria* and *Bacillus* were greater than 1% of the rhizosphere and rhizosphere, respectively (Fig. S5). Thus, the dominant taxa *K. konosiri* 1-1 and *B. marisflavi* 23-1, with high abundance and strong growth-promoting function, were selected for coculture with rice seedlings to observe the growth-promoting effect on rice seedlings under salt treatment and water treatment. The results showed that *K. konosiri* can significantly promote shoot length (***, *P* < 0.001), root length (*, *P* < 0.05), and total length (***, *P* < 0.001) of the rice seedlings under 100 mmol · L^−1^ NaCl treatment, while it can greatly promote root development under water conditions (*, *P* < 0.05) (Fig. 6d and f). Similarly, *B. marisflavi* can significantly promote shoot length, root length, and total length of the rice seedlings under 100 mmol · L^−1^ NaCl treatment (***, *P* < 0.001), while it can greatly promote shoot development and overall length under water treatment (*, *P* < 0.05) (Fig. 6e and g). Overall, it can be seen that the addition of the two strains can significantly promote the growth and development of rice seedlings under both salt and water conditions.

## DISCUSSION

### Salinity had different effects on phyllosphere and rhizosphere microbial communities and had a greater impact on bacterial communities.

We explored the differences in the effects of salinity on the microbial communities in the phyllosphere and rhizosphere of *A. marina* in the three salinity gradients. The results showed that the alpha diversity of the rhizosphere bacterial community increased significantly with increasing salinity (Fig. 2a). This is consistent with a previous study, which showed that bacterial alpha diversity was high during the low-salinity period in coastal ecosystems (15). It has also been reported that leaf salt secretion is enhanced with increasing soil salinity (13). This indicates that the increase in rhizosphere salinity will lead to an increase in phyllosphere salinity, which will filter out some microorganisms, and fewer microorganisms adapted to harsh environments migrate from the rhizosphere to the phyllosphere. However, there was no significant difference in the alpha diversity of phyllosphere bacteria with increasing salinity (Fig. 2a). This might be because the small-amplitude salinity change had not reached the community salinity tolerance threshold for the phyllosphere of *A. marina* (16). It also reflects that the changes in soil salinity in the phyllosphere and rhizosphere are not consistent.

The beta diversity of phyllosphere and rhizosphere microbial communities was significantly separate under the three salinities. This result confirmed that salinity has important effects on the microbial community structure (17, 18). Moreover, the effects of salinity on microbial community composition were significantly different between the phyllosphere and rhizosphere. For example, the relative abundance of *Proteobacteria* decreased significantly with increasing salinity, while the relative abundance of *Bacteroidota* increased in the phyllosphere bacterial community (Fig. 3d). This is not exactly the same finding as that of other studies, which reported that the relative abundance of *Bacteroidota* and *Proteobacteria* was higher under high-salinity conditions in coastal farmland (19). Although *Proteobacteria* are common bacteria with strong salt tolerance (19), it has been reported that *Betaproteobacteria* are greatly inhibited in saline environments, thus resulting in a significant decrease in phyllosphere *Proteobacteria* with increasing salinity. This may be the cause of the presence of salt-sensitive *Betaproteobacteria*. On the other hand, *Bacteroidota* are associated with high salinity in aquatic environments (20) and can minimize salt absorption through specific membrane or cell wall structures (21). Moreover, the relative abundance of the *Zunongwangia* genus in *Bacteroidota* also increased with salinity, and most members of this genus can secrete a large amount of exopolysaccharides, which may help the microbial community tolerate extreme salinity and nutrient availability (22). Therefore, the relative abundance of *Bacteroidota* increases with increasing salinity, which also reflects the high growth rate and adaptation of *Bacteroidota* in high salt. In the rhizosphere bacterial community, the relative abundance of *Actinobacteriota* increased significantly with increasing salinity (Fig. 3b). This finding ties well with previous studies in which salinity is positively correlated with the diversity of the actinobacterial community (23). Additionally, many *Actinobacteriota* members can degrade complex compounds, such as polysaccharides and phenolic compounds (24), which allows them to obtain nutrients and survive in a more barren and hostile environment. Salinity caused less disturbance to fungal communities than to bacterial communities. Only the relative abundance of Dothideomycetes decreased significantly in the phyllosphere and rhizosphere with increasing salinity (Fig. 3c and d). These results indicated that the fungal community was more stable in the salt environment.

### Response of microbe network structure to salinity in the phyllosphere and rhizosphere of *A. marina*.

It has been reported that salinity filtration is one of the ecological processes driving species symbiosis patterns (25) and has an impact on the complexity and stability of microbial communities (26, 27). By studying the bacterial phyla and fungal classes with the highest connectivity, which are generally considered to be the keystone taxa in a network, we found that the microbial network structure in the phyllosphere and rhizosphere responded differently to increasing salinity, and the fungal network interaction was basically not affected by salinity. At the same time, the increase in rhizosphere salt will promote the secretion of salt in the phyllosphere and further lead to an increase in *Bacteroidota* abundance, which is related to the good nutritional flexibility and stress response ability of *Bacteroidota* (28). The rhizosphere is a more complex environment. The increase in salinity reduces the abundance of some salt-sensitive *Proteobacteria*, while *Actinobacteriota* with strong salt tolerance also increase significantly with salinity. This is related to the ability of *Actinomycetes* to produce large amounts of extracellular polymers and active substances, making them more able to cope with nutrient deficiencies and salt stress. This result suggested that the bacterial network structure in both the phyllosphere and rhizosphere responds to increasing salinity by increasing the abundance of taxa with strong salt tolerance and nutrient utilization, such as *Bacteroidota* and *Actinobacteriota*.

One of the most useful features of network analysis is “hubs,” taxa that are highly connected nodes in a microbiome (29). These hubs have been proposed as keystone taxa, as their removal would cause a drastic shift in the composition and functioning of a microbiome (30). Thus, we explored the effects of different salinities on the highly interconnected hub microbial taxa known as “nodes of maximum degree” in the phyllosphere and rhizosphere communities. The results showed that in the phyllosphere bacterial network, the nodes of maximum degree that belonged to the *Alphaproteobacteria* changed significantly with increasing salinity. Changes in salinity had a greater impact on the stability of phyllosphere bacteria, and the bacterial community maintained stability in a high-salt environment by regulating the abundance of key taxa.

### With a change in salinity, the functions of microbial communities in the phyllosphere and rhizosphere were adjusted adaptively.

The plant microbiome is known as the second genome of plants (31), and plant-related microbial communities play an irreplaceable role in plant resistance to stress (32, 33). Our results showed that salt stress increased the activity of enzymes associated with stress resistance, particularly catalase, in the phyllosphere and rhizosphere bacterial microbial communities (Fig. 5a and b). In fungal communities, the abundance of catalase and tryptophan synthase increased significantly with increasing salinity in the phyllosphere, but they did not change significantly in the rhizosphere (Fig. 5c and d). More than 60% of the fungi in the fungal community belong to Ascomycota, the cell walls of which are rich in melanin. Thus, salinity has little influence on the fungal community because high proportions of Ascomycota can resist various abiotic stresses (34). Meanwhile, previous studies have shown that salinity can affect community functions such as microbial decomposition processes (35), organic carbon decomposition, and biogeochemical cycles of carbon (36), nitrogen (37), and phosphorus (38). Soil microbes are the key driving factors of phosphorus transformation and dominate the composition of phosphorus forms (39), and alkaline phosphatase has a high capacity to mineralize organophosphorus compounds in soil (40). In this study, we found that with increasing salinity, the relative abundance of potential alkaline phosphatase in the phyllosphere and rhizosphere bacterial communities increased, which is consistent with the positive correlation between increased salinity and alkaline phosphatase activity reported by Morrissey et al. (41).

Finally, the dominant bacteria *Kushneria* and *Bacillus* were isolated from the phyllosphere and rhizosphere, respectively. Their coculture with salt-stressed rice could effectively alleviate salt stress and promote the growth of rice. *Bacillus* is a relatively common plant growth-promoting rhizobacterium (PGPR) (42, 43), and *Kushneria* is also a growth-promoting bacterium (44). Interestingly, we found that *Bacillus* was the dominant species only in the rhizosphere environment and hardly existed in the phyllosphere, while *Kushneria* was the dominant species only in the phyllosphere (see Fig. S4 in the supplemental material). This may be a compensation for the emergence of functional microorganisms in the process of adapting to the environment in different plant niches. In the context of sustainable agriculture, it is of practical significance to use salt-tolerant microorganisms to achieve salt tolerance and yield increases in crops.

In conclusion, we investigated the effects of different salinities on the microbial community structure, function, and microbial interaction network between the phyllosphere and rhizosphere of *A. marina*, the most salt-tolerant plant in the mangrove ecosystem. The results showed that salinity had different effects on the assembly of microbial communities in the phyllosphere and rhizosphere and had greater effects on the diversity of bacterial communities than fungal communities. With increasing salinity, the relative abundance of *Proteobacteria* decreased, while that of *Bacteroidota* and *Actinobacteriota* increased significantly in the phyllosphere bacterial community. This is a strategy for bacterial communities to maintain community stability in high-salt environments by regulating the abundance of key groups. Moreover, the abundance of catalase and enzymes associated with phosphorus utilization increased significantly in the phyllosphere and rhizosphere bacteria in response to increasing salinity. Finally, important functional bacteria were isolated and obtained. Future work may be focused on the mechanism concerning the salt tolerance and growth promotion characteristics of these microbial resources and their application in agriculture.

## MATERIALS AND METHODS

### Study site, sampling, and environmental information.

In this study, sampling was conducted in the Zhanjiang Mangrove National Nature Reserve and Shenzhen Bay, both located in Guangdong Province of China. *A. marina* samples were collected at three sites, Taiping Town, Leizhou, and Shenzhen (see Fig. S7 in the supplemental material for detailed locations). All samples were collected daily from 27 March 2021 to 31 March 2021. Located in the southernmost part of mainland China, the Zhanjiang Mangrove National Nature Reserve (20°14′ to 21°35′N, 109°40′ to 110°35′E) is intermittently distributed along the coastline of the Leizhou Peninsula. It has a tropical monsoon climate that is mild and humid with an average temperature of 23.4°C and an annual average precipitation of 1,800 to 2,000 mm (45). The Shenzhen Bay Mangrove Wetland (22°30′ to 22°39′N, 113°53′ to 114°05′E) covers an area of approximately 100 km^2^. It has a subtropical marine climate with an average annual temperature of 22.4°C and an average annual rainfall of 1,700 to 1,900 mm (46).

Leaves and finger-like respiratory roots of *A. marina* were collected from Shenzhen Bay, Taiping, and Leizhou. Leaf sampling methods refer to the protocol reported by Yao et al. (45). In brief, at each sampling site, 30 healthy leaves were randomly selected from *A. marina* and adjacent *A. marina*, with 3 replicates in each region (distance between replicates, >50 m). Meanwhile, finger-like roots and soil of *A. marina* were collected by a five-point sampling method, the soil sampling range was within 5 to 20 cm from the ground, and the samples were mixed well (47). Samples were placed in sterile bags and ice boxes. Some samples were used for culturable microbial screening, and the rest of the samples were stored at −80°C for DNA extraction.

### SEM analysis.

The colonization of microorganisms on leaves was observed by SEM. Sterilized scissors were used to cut 5-mm by 5-mm mature leaf tissue pieces of *A. marina* (approximately 10 pieces from each sampling site), the front side was marked, and the pieces were placed in precooled 2.5% glutaraldehyde solution. The samples were immediately vacuumized until the blade sank into the glutaraldehyde solution (48). The fixed samples were washed in sequence with phosphate buffer (0.1 mol · L^−1^, pH 7.4) and 30%, 40%, 50%, 70%, 90%, and 100% (vol/vol) ethanol for 15 min each. The dehydrated samples were dried at the critical point of CO_2_ and then sprayed with a vacuum ion sputtering apparatus for 15 s (49). Finally, the microstructure and microbial colonization of leaves were observed under an SEM (model S-3400N; Hitachi, Japan).

### Collection of microbial samples from phyllosphere and rhizosphere.

The leaf samples were treated as described by Yao et al. (50) with some modifications. Briefly, each sample of 3 g was randomly weighed and placed in a 50-mL sterile plastic tube. Then, 30 mL of sterile phosphate-buffered saline (PBS) (0.1 mol · L^−1^, pH 7.4) was added, followed by ultrasound for 1 min alternating with vortexing for 30 s (repeated 3 times). Then, the leaves were moved into a new 50-mL sterile plastic tube, and the above-described steps were repeated. The above two supernatants were mixed and centrifuged at 13,000 rpm for 10 min to collect the precipitate. The rhizosphere samples were subjected to the method reported by Edwards et al. (51). The bulk soil was first shaken off on the ultraclean bench, leaving the root system attached to the soil approximately 1 mm thick at the root. The roots and rhizosphere soil were transferred to a 50-mL sterile plastic tube containing 30 mL sterile PBS (0.1 mol · L^−1^, pH 7.4) solution and placed in a shaker for 20 min (150 rpm). The root and rhizosphere soil were transferred to a 50-mL sterile plastic tube containing 30 mL sterile PBS (0.1 mol · L^−1^, pH 7.4) solution and placed on a shaker for 20 min (150 rpm). Roots were removed with sterile forceps and centrifuged for 20 min at 13,000 rpm to obtain the precipitate of the suspension. All processed samples were stored at –80°C until DNA extraction.

### DNA extraction and PCR amplification.

The total DNA was extracted from phyllosphere and rhizosphere samples using the E.Z.N.A. soil DNA kit (Omega Biotek, Norcross, GA, USA). The DNA extract was examined using a 1% agarose gel, and the DNA concentration and purity were measured by a NanoDrop 2000 UV-Vis spectrophotometer (Thermo Scientific, Wilmington, DE, USA). Primers 338F (5′-ACTCCTACGGGAGGCAGCAG-3′) and 806R (5′-GGACTACHVGGGTWTCTAAT-3′) (52) were used to amplify the V3-V4 region of the bacterial 16S rRNA gene, and primers ITS1F (5′-CTTGGTCATTTAGAGGAAGTAA-3′) and ITS2R (5′-GCTGCGTTCTTCATCGATGC-3′) were used to amplify the ITS1 region of the fungal rRNA gene (53). PCR amplification was carried out on an ABI GeneAmp 9700 PCR thermocycler (ABI, CA, USA), and the steps were as follows: DNA predenaturation at 95°C for 3 min, followed by 27 cycles (16S rRNA)/37 cycles (ITS1 rRNA) of denaturing at 95°C for 30 s, annealing at 55°C for 30 s, and extension at 72°C for 45 s, and a single extension at 72°C for 10 min. Each sample was amplified in a 20-μL reaction system containing 4 μL of 5 × TransStart FastPfu buffer, 2 μL of deoxynucleoside triphosphates (dNTPs), 0.8 μL of forward primer (5 μM), 0.8 μL of reverse primer (5 μM), 0.4 μL of TransStart FastPfu DNA polymerase, 10 ng of template DNA, and double-distilled water (ddH_2_O). PCRs were performed in triplicate. PCR products were detected using a 2% agarose gel, purified using an AxyPrep DNA gel extraction kit (Axygen Biosciences, Union City, CA, USA), and quantified using a Quantus fluorimeter (Promega, USA).

### Illumina MiSeq sequencing.

Purified amplicons were pooled in equimolar amounts and paired-end sequenced on an Illumina MiSeq PE300 platform (Illumina, San Diego, CA, USA) according to the standard protocols by Majorbio Bio-Pharm Technology Co., Ltd. (Shanghai, China).

### Bioinformatics analysis.

The raw 16S rRNA and fungal internal transcribed spacer (ITS) sequencing were quality filtered by FASTQ version 0.20.0 (54) and merged by FLASH version 1.2.7 (55). We removed low-quality reads with an average quality score below 30, no valid primer sequence or barcode sequence, ambiguous bases, or length under 200 bp. The paired 16S rRNA/ITS sequences were merged into a single sequence.

After identification and deletion of chimeric sequences, OTUs with a sequence similarity cutoff of 97% were clustered using UPARSE version 7.1 (56). RDP version 2.2 was used to classify the OTU representative sequences of bacteria and fungi from the 16S rRNA database Silva V138 and ITS database Unite8.0, respectively, with a confidence threshold of 0.7 (57). All OTUs identified as belonging to “chloroplast” and “mitochondria” were deleted from the 16S rRNA data set. Finally, to avoid the potential impact of different sequence numbers in the samples on the identified bacterial/fungal communities, MOTHUR version 1.31.2 was used to normalize the sequence number of each sample to a minimum sample size (58).

### Network construction and analysis.

Random matrix theory (RMT)-based molecular ecological networks provide a powerful tool for elucidating cooccurrence patterns in microbial communities and for analyzing their responses to environmental changes. In this study, RMT was used to construct microbial interaction networks. Phyllosphere and rhizosphere bacterial and fungal networks, as well as phyllosphere and rhizosphere bacterial-fungal interaction networks, were constructed. A publicly available Molecular Ecological Network Analysis Pipeline (MENA; http://ieg2.ou.edu/MENA/) was used to construct the network (59). Only OTUs detected in each set of more than 5 replicate samples were retained in network construction. RMT was used to automatically identify an appropriate similarity threshold (St) before network construction (24), and each network directly uses the appropriate threshold automatically detected by the system. The Spearman coefficient was used to calculate the correlation. The networks were visualized using Gephi 0.9.2 (60).

Soil physicochemical properties were analyzed according to the protocols of Zhang et al. (18). In brief, soil pH was determined using a 1:2.5 soil-water mixture by a pH meter with a glass electrode (PHS-320). Soil EC was determined using a 1:5 soil-water mixture by an EC meter (DDS-307A). Total nitrogen (TN) was quantified by using the automatic nitrogen analyzer method, and total carbon (TC) was analyzed on an elemental analyzer. Soil organic matter (SOM) was determined by using the method of potassium dichromate oxidation with external heating. In addition, salinity was measured *in situ* in soil surface water or tidal flat water around *A. marina* by using a portable salinity meter (Model: AZ8371, AZ Instrument Corp.). For results on the physicochemical parameters of rhizosphere soil samples from three sampling sites of *A. marina*, see Table S2.

### Acquisition of culturable microorganisms and growth-promoting experiments on rice seedlings.

Phyllosphere and rhizosphere microbial samples were obtained according to the above-described method. The 10^−4^ samples were selected and cultured on R_2_A (R_2_A medium developed by Reasoner and Geldreich is a low nutrient medium) and peptone-dextrose agar (PDA) medium. The rhizosphere samples were also coated by the gradient dilution method. The culturable strains were purified and cultured, and phosphorus solubilization, nitrogen fixation, potassium solubilization, and salt tolerance were qualitatively determined. Combined with high-throughput sequencing data, the strains with higher abundance in the phyllosphere and rhizosphere were selected to verify the growth-promoting function of rice seeds under salt stress, including Kushneria konosiri 1-1 in the phyllosphere and Bacillus marisflavi 23-1 in the rhizosphere (Fig. S4). The two strains were shaken and activated with R_2_A liquid medium, and the precipitated bacteria were collected by centrifugation and washed twice. Sterile water and 100 mmol · L^−1^ NaCl solution were used in the experimental procedure. The bacteria were suspended in sterile water or 100 mmol · L^−1^ sterile NaCl solution, and then the OD_600_ was adjusted to 0.2, while the seeds in the control group were treated with the same amount of sterile water or 100 mmol · L^−1^ sterile NaCl. Healthy, plump rice seeds were selected for routine surface disinfection. Each treatment was repeated 3 times, and 10 mL of bacterial suspension or sterile water was added to each petri dish. Rice seed germination and seedling growth were observed after inoculation of *K. konosiri* strain 1-1 and *B. marisflavi* strain 23-1 suspensions at 12 and 8 days, respectively.

### Statistical analysis.

Statistical analysis was performed using R software (v3.3.1). The rarefaction curve-based OTU level associated with the phyllosphere and rhizosphere was determined using the specaccum function in the vegan package. RDA and CCA were used to assess the relationship between soil physicochemical properties and the rhizosphere bacterial community and fungal community, respectively, at the genus level. The significance of CCA or RDA was judged by permutest analysis. Analysis and mapping of RDA and CCA were performed using the vegan package in R (v3.3.1). Mantel tests were used to correlate the distance matrices between soil bacterial communities (weighted UniFrac dissimilarity) with environmental factors (Bray-Curtis distance) (61). The alpha diversity indices (Shannon and Chao1) were calculated using the vegan package in R (62). PCoA based on Bray-Curtis distance was used to visualize dissimilarity in bacterial and fungal communities, and then analysis of variance (ANOVA) was performed using distance matrices (Adonis) according to three salinity gradients using 999 permutations (63). PICRUSt2 software was used to predict the relative abundance of related enzymes based on the taxonomy of 16S rRNA gene sequences (64) and fungal ITS gene sequences (62). Student's *t* test was used to compare the two groups (58), and one-way ANOVA with Tukey’s honestly significant difference (HSD) was used to compare the three salinity gradients (2).

### Data availability.

Raw sequence data for 16S rRNA and ITS reads were deposited into the NCBI Sequence Read Archive under BioProject number PRJNA860025. The 16S rRNA gene sequences of strains Kushneria konosiri 1-1 and Bacillus marisflavi 23-1 in this study were deposited in GenBank under accession numbers ON203058 and ON203056, respectively.
