# Supplementary material for: Effects of Salinity on Assembly Characteristics and Function of Microbial Communities in the Phyllosphere and Rhizosphere of Salt-Tolerant Avicennia marina Mangrove Species
Source: Microbiol Spectr. 2023 Feb 6;11(2):e03000-22. doi: 10.1128/spectrum.03000-22 (PMC10101020; doi:10.1128/spectrum.03000-22)
Supplement: Supplemental file 1 — Supplemental material. Download spectrum.03000-22-s0001.pdf, PDF file, 1.7 MB [file spectrum.03000-22-s0001.pdf]

Effects of salinity on assembly characteristics and function of microbial communities in the phyllosphere and rhizosphere of salt-tolerant *Avicennia marina* mangrove species

Authors:

Xiangxia Yang[a], Zhian Dai[a], Rongwei Yuan[a], Zhenhua Guo[a], Hanxiao Xi[a], Zhili He[b], Mi Wei[a,*]

Affiliation:

[a] School of Agriculture, Shenzhen Campus of Sun Yat-sen University, Shenzhen 518107，Guangdong, China.

[b] Southern Marine Science and Engineering Guangdong Laboratory (Zhuhai), Zhuhai 519080, China.

*** Correspondence**

E-mail: weim29@mail.sysu.edu.cn

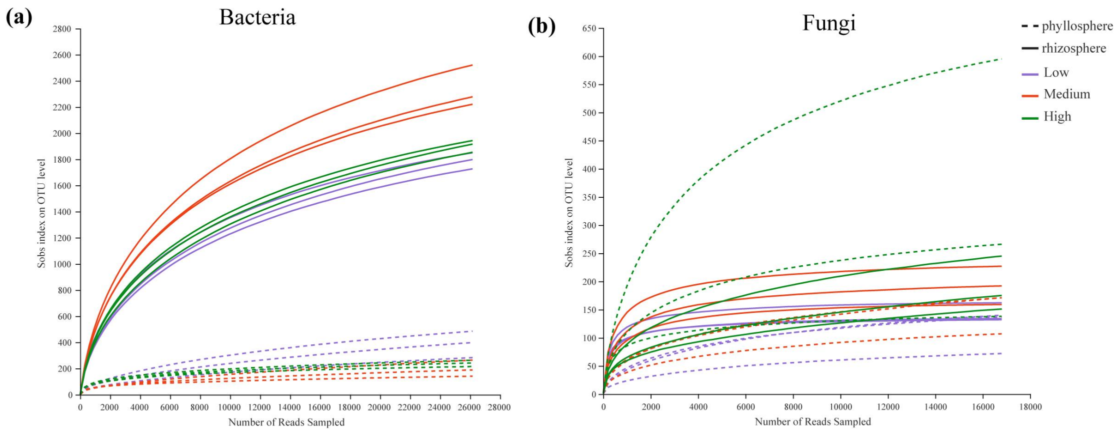

Fig. S1 Rarefaction curves of the phyllosphere and rhizosphere: (a) bacteria and (b) fungi. Rarefaction curves were assembled showing the number of OTUs relative to the number of total sequences.

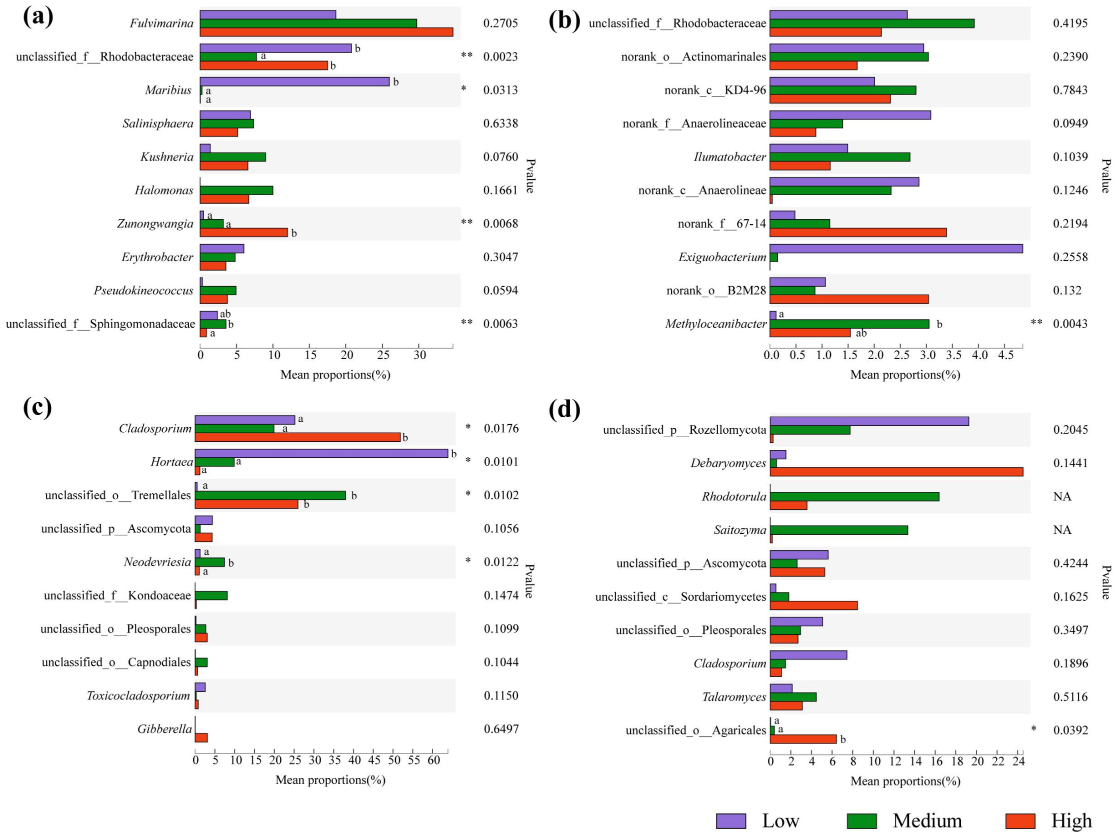

Fig. S2 The top 10 relative abundances of bacterial and fungal taxa at the genus level under three salinity gradients. (a) phyllosphere bacteria, (b) rhizosphere bacteria, (c) phyllosphere fungi, (d) rhizosphere fungi. The X-axis represents the average relative abundance (n=3) in different groups of species, and the columns with different colors represent different groups. Different lowercase letters

indicate significant differences (ANOVA, Tukey's HSD test, P < 0.05) among the three salinity gradients. On the far right is the P value, *P < 0.05, **P <0.01, ***P <0.001.

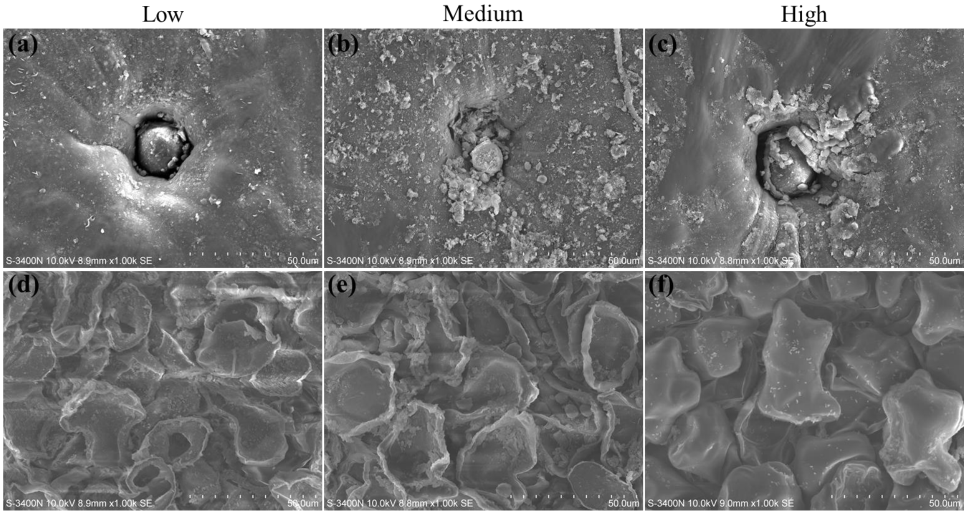

Fig. S3 Microscopic images of microorganism colonization on leaf surfaces of *A. marina.* (a), (b), and (c) are the front sides of the leaves of *A. marina*, and (d), (e), and (f) are the reverse surfaces of the leaves of *A. marina*.

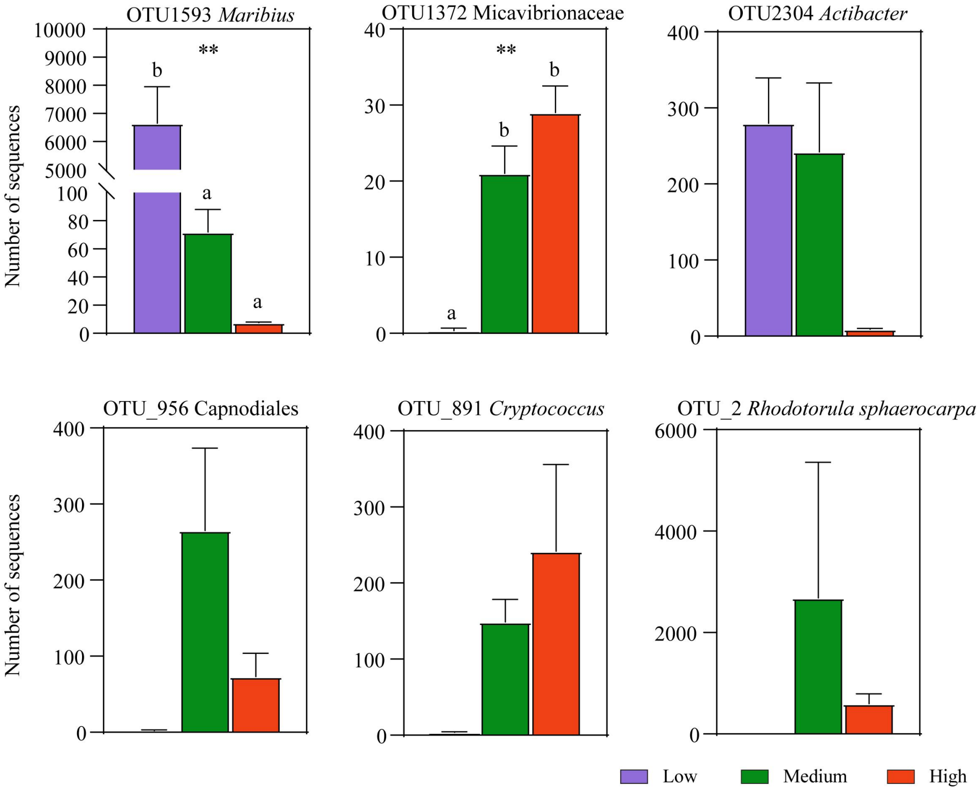

Fig. S4 Absolute abundance of nodes with maximum degree in the phyllosphere and rhizosphere networks. Data are the mean ± standard error (n=3); different lowercase letters indicate significant differences (ANOVA, Tukey's HSD test, $P < 0.05$) among the three salinity gradients, *$P < 0.05$, **P <0.01, ***P <0.001.

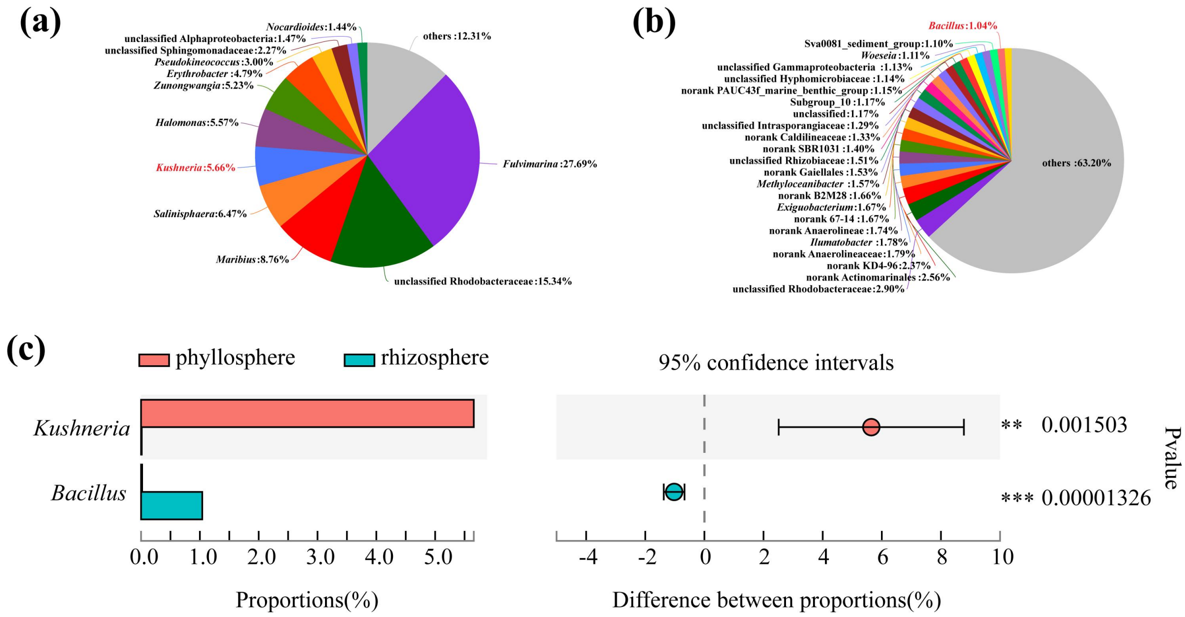

Fig. S5 Relative abundance of bacteria and fungi at the genus level in the phyllosphere and rhizosphere microbial communities of *A. marina*. (a) phyllosphere bacteria, (b) rhizosphere bacteria. Bacterial genera accounting for < 1% of total reads were assigned to 'others'. (c) Relative abundance of *Kushneria* and *Bacillus* in phyllosphere and rhizosphere microbial communities of *A. marina*. The bar charts show the differences between the proportions of sequences in each group with a confidence interval of 95%, two-tailed Student's t test with FDR testing correction, *P< 0.05, **P <0.01, ***P <0.001.

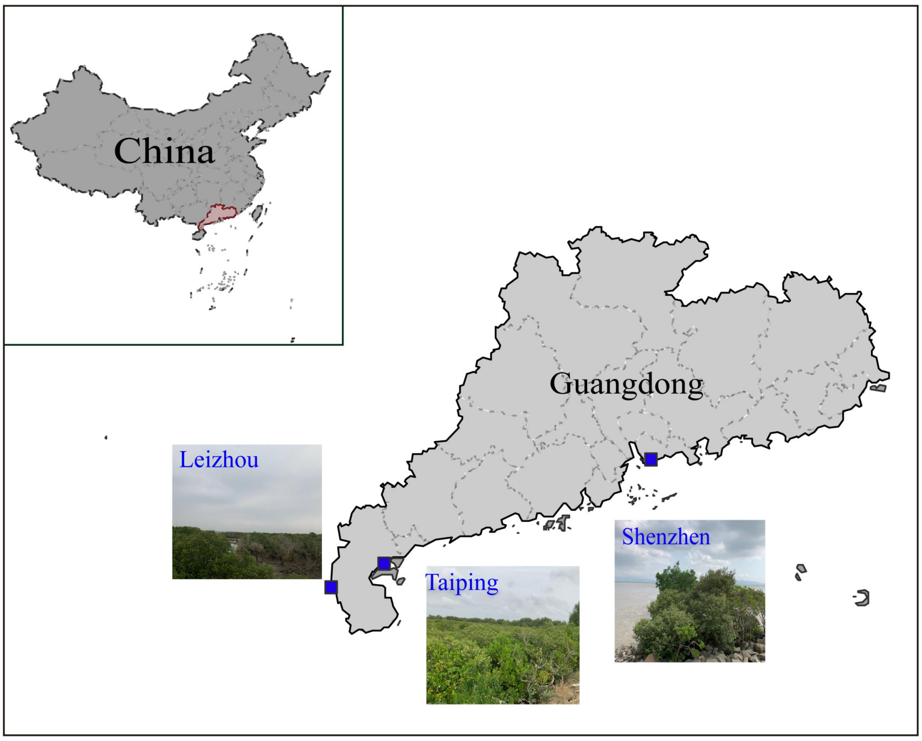

Fig. S6 Overview map of the *Avicennia marina* sampling area for this study.

Table S1 High-throughput sequencing data statistics of microbial communities in the *A. marina* phyllosphere and rhizosphere.

Sample group	Number of optimized sequences	Sequence numbers of per sample （Normalized）	Phylum	Class	Order	Family	Genus	Species	OTU
**Bacteria**									
phyllosphere	565940	26159	34	81	189	300	473	689	1016
rhizosphere	500233	26159	54	156	386	610	1065	1877	4909
**Fungi**									
phyllosphere	642361	16801	5	23	55	121	205	283	500
rhizosphere	530465	16801	10	34	85	193	367	557	1144

Table S2 Soil physicochemical properties of three sampling sites of *Avicennia marina*.

Sample group	EC ($mS\cdot cm^{-1}$)	Salinity (ppt)	pH	TN ($g\cdot kg^{-1}$)	TC ($g\cdot kg^{-1}$)	SOM ($g\cdot kg^{-1}$)	Salinity level
Shenzhen	1.76 ± 0.07^{a}	10.52 ± 0.39^{a}	7.67 ± 0.01^{b}	0.33 ± 0.01^{a}	14.03 ± 0.24^{a}	20.99 ± 0.24^{a}	Low
Leizhou	2.72 ± 0.08^{a}	26.97 ± 1.20^{b}	7.62 ± 0.11^{b}	0.90 ± 0.11^{b}	23.47 ± 1.46^{b}	36.45 ± 2.40^{b}	Medium
Taiping	5.60 ± 0.30^{b}	33.43 ± 1.52^{b}	7.01 ± 0.08^{a}	0.78 ± 0.02^{b}	22.67 ± 0.49^{b}	34.74 ± 0.80^{b}	High

Note: Data are the mean ± standard error (n=3); Different lowercase letters indicate significant differences (ANOVA, Tukey's HSD test, $P < 0.05$) among groups. EC, soil electrical conductivity; SOM, soil organic matter; TN, total nitrogen; TC, total carbon.

Table S3 Mantel test between rhizosphere bacterial/fungal communities based on Bray–Curtis dissimilarity with environmental factors.

	Bacterial community		Fungal community	
	r	*p*	r	*p*
EC	0.6652	**0.001**	0.4543	**0.012**
Salinity	0.5262	**0.006**	0.5134	**0.012**
pH	0.3438	**0.037**	0.1821	0.347
SOM	0.4548	**0.008**	0.4707	**0.006**
TN	0.4705	**0.015**	0.5284	**0.005**
TC	0.4585	**0.012**	0.4733	**0.004**

Note: The r indicates Spearman's rank correlation coefficient. Significance is marked in bold ($P < 0.05$).
